# Hepatic irradiation persistently eliminates liver resident NK cells

**DOI:** 10.1371/journal.pone.0198904

**Published:** 2018-06-13

**Authors:** Ryosuke Nakano, Masahiro Ohira, Takuya Yano, Yuki Imaoka, Yuka Tanaka, Hideki Ohdan

**Affiliations:** 1 Department of Gastroenterological and Transplant Surgery, Applied Life Sciences, Institute of Biomedical & Health Sciences, Hiroshima University, Hiroshima, Japan; 2 Division of Regeneration and Medicine, Medical Center for Translational and Clinical Research, Hiroshima University Hospital, Hiroshima, Japan; Western Sydney University, AUSTRALIA

## Abstract

Hepatic irradiation for the treatment of hepatobiliary malignancies often indirectly damages liver tissue and promotes the development of liver fibrosis. However, little is known concerning the effects of hepatic irradiation on the liver immune system, including natural killer (NK) cells. The aim of this study was therefore to investigate how hepatic irradiation influences the functions and characteristics of liver resident NK cells. An established murine hepatic irradiation model was used to examine the specific effects of hepatic irradiation on immune cell populations and metastasis. This analysis demonstrated that hepatic irradiation decreased the number of liver resident NK cells (DX5^–^TRAIL^+^), but did not affect the total NK number or proportions of NK cells in the liver or spleen. This effect was correlated with the hepatic irradiation dose. Surprisingly, the liver resident NK population had not recovered by two months after hepatic irradiation. We also found that hepatic irradiation limited the cytotoxic effects of liver-derived lymphocytes against a mouse hepatoma cell line and promoted hepatic metastases in an *in vivo* model, although adoptive transfer of activated NK cells could alleviate metastatic growth. Finally, we demonstrated that hepatic irradiation disrupted the development of liver-resident NK cells, even after the adoptive transfer of precursor cells from the bone marrow, liver, and spleen, suggesting that irradiation had altered the developmental environment of the liver. In summary, our data demonstrated that hepatic irradiation abolished the DX5^–^TRAIL^+^ liver-resident NK cell population and dampened antitumor activities in the liver for at least two months. Additionally, hepatic irradiation prevented differentiation of precursor cells into liver-resident NK cells.

## Introduction

Hepatobiliary malignancies are a challenging medical issue due to high incidence rates and relatively aggressive behavior. Although surgical resection is the standard method of treatment, some patients are inoperable at the point of presentation. To counter this, use of radiation therapy, including stereotactic body radiation therapy and hypofractionated proton therapy, has gradually increased and continues to improve [1]. However, the liver is often incidentally irradiated during radiation therapy for tumors [2]. Subsequent damage to tissues ultimately culminates in fibrosis due to the release of various pro-fibrogenic cytokines, including platelet-derived growth factor (PDGF) and TGF-β [3]. Radiation can also affect the immune environment. For example, radiation treatment leads to a marked increase in CXCL16 secretion by breast tissue, promoting the recruitment of effector T cells to sites of inflammation in mice [4]. The direct lymphocyte response to radiation exposure is highly variable and natural killer T (NKT) and regulatory T cells are both relatively radio-resistant compared to other lymphocyte populations in humans [5, 6]. However, the effect of hepatic irradiation on liver resident natural killer (lrNK) cells remains unclear. Therefore, we intended to analyze the effect of hepatic irradiation on the phenotype, function, and development of liver resident NK cells in this study.

NK cells are a particularly important cell population that play a key role in the innate immune response, possessing both cytotoxic and cytokine-producing effector functions that act as a first line of defense against disease [7]. The liver contains a relatively large number of NK cells that are phenotypically and functionally distinct from other circulating NK cells (cNK cells) in humans [8, 9]. For example, lrNK cells can be defined as DX5^–^CD49a^+^TRAIL^+^ CD69^+^ and CXCR6^+^ in mice [10]. They have crucial functions in defending against viral infections [11, 12], tumors [8, 13, 14], and possess memory-like properties [15].

Despite the relative importance of lrNK cells, their response to irradiation is unclear. To examine this in more detail, the present study investigated the influence of hepatic irradiation on the function and phenotypes of lrNK cells using a mouse hepatic irradiation model. We also assessed whether liver irradiation affected the differentiation of NK cells in the liver. Our data demonstrate that hepatic irradiation specifically eliminated the lrNK cell population for at least two months and that this deficit was not restored by adoptive transfer of precursor cells. We also found that hepatic irradiation limited the cytotoxic properties of liver-derived lymphocytes and affected the progression of metastases *in vivo*. However, adoptive transfer of activated NK cells was able to limit the rate of metastasis. These data will have important implications for the future treatment of hepatobiliary malignancies with irradiation and underscore the damage that such treatments can have on the immune system.

## Materials and methods

### Mice

C57BL/6J (B6) (H-2b) mice were purchased from CLEA Japan, Inc. (Osaka, Japan). RAG-2γ(c) knockout (C57BL/6J × C57BL/10SgSnAi[KO]γc[KO]Rag-2) mice were obtained from Taconic Farms (Hudson, NY, USA). The mice were housed in the animal facility of Hiroshima University, Japan, in a pathogen-free microenvironment. Both male and female mice were used between 8 and 12 weeks of age. Mice were euthanized by cervical dislocation after isoflurane inhalation, when indicated. All efforts were made to minimize the suffering of animals for the duration of their lives and during sacrifice.

### Ethics statement

This study was performed in strict accordance with the Guide for the Care and Use of Laboratory Animals and the local committee for animal experiments. The experimental protocol was approved by the Ethics Review Committee for Animal Experimentation of the Graduate School of Biomedical Sciences, Hiroshima University (Permit Number: A17-60). All animal experiments were performed according to the guidelines set out by the US National Institutes of Health (1996). This work was performed in part at the Research Facilities for Laboratory Animal Science, Natural Science Center for Basic Research and Development (N-BARD), Hiroshima University, Japan.

### Hepatic irradiation

Whole-liver irradiation was performed as previously described [16, 17]. Briefly, before irradiation, each animal was anesthetized by intraperitoneal injection of xylazine (5 mg/kg body weight) and ketamine (100 mg/kg body weight) and the abdomen was surgically opened. A 3-mm-thick high-density tungsten sheet (density: 12, Nippon Tungsten Co. Ltd, Fukuoka, Japan) was placed behind the liver to shield the gastrointestinal tract before irradiation of supine-positioned mice. Whole-liver irradiation was delivered by a single anterior beam using a 320 MGC Philips orthovoltage unit operating at 320 kVP, 10 mA, and 0.5-mm copper filtration (Phillips, Amsterdam, Netherlands). The dose rate was 320 cGy/min to the midline at a 2-cm depth within the jig at a 35-cm source-to-surface distance. The blood samples were obtained from the caudal vein for measurement of aspartate aminotransferase (AST) and alanine aminotransferase (ALT) levels as markers of liver damage; all blood tests were performed at FUJIFILM Monolith Co., Ltd. (Tokyo, Japan). For the histological evaluation of the liver, 4-µm tissue sections were stained with hematoxylin and eosin (HE). Mice were sacrificed 2, 4, 6, or 8 weeks post irradiation for investigation.

### Isolation of lymphocytes

Lymphocytes were isolated from the liver, spleen, and bone marrow (BM) of untreated and irradiated mice under anesthesia. Liver lymphocytes were prepared as previously described [18]. Briefly, after injection of 1 mL of phosphate-buffered saline (PBS) supplemented with 10% heparin into the portal vein, the liver was resected and perfused with 50 mL of PBS supplemented with 0.1% ethylenediaminetetraacetic acid (EDTA). Blood cells were harvested from the liver perfusate by centrifugation and erythrocytes were removed using an ammonium chloride potassium lysing buffer. The isolated spleen isolated was mechanically dissociated, and then erythrocytes were lysed using an ammonium chloride potassium lysing buffer. We obtained BM cells by flushing femurs and then lysing erythrocytes using the ammonium chloride potassium lysing buffer.

### Flow cytometric analysis

Flow cytometric analyses were performed using a FACSCanto II cytometer (BD Biosciences, Mountain View, CA, USA) or the LSRFortessa X-20 system (BD Biosciences, Mountain View, CA, USA). For phenotyping of NK cell surface markers, liver, spleen and BM lymphocytes were stained with anti-NK1.1 (PK136), anti-CD69 (H1.2F3), and anti-CD3(145-2C11), (both BD Biosciences, Mountain View, CA, USA), anti-TCRβ chain (H57-597), anti-TRAIL (CD253) and anti-TCRγδ chain (GL3) mAbs (both BioLegend, San Diego, CA, USA). Nonspecific FcγR binding of labeled mAbs was blocked using an anti-CD16/32 mAb (2.4G2; BD PharMingen, Hamburg, Germany). Dead cells were excluded from the analysis by forward scatter and propidium iodide (PI; Sigma-Aldrich, St. Louis, MO, USA), or 7-amino-actinomycin D(7-AAD; BD Biosciences, Mountain View, CA, USA) staining.

### Hepatoma cell line

The mouse hepatoma cell line Hepa1-6 (derived from H-2b mice) was purchased from RIKEN Cell Bank (Tsukuba, Japan).

### Cytotoxicity assays

Cell cytotoxicity was assessed as previously described [13]. Briefly, Hepa1-6 cells were labeled with Na_2_[^51^Cr]O_4_ for use as target cells and then incubated with effector cells in round-bottomed 96-well plates for 4 h at 37°C. The percent cytotoxicity, as indicated by ^51^Cr release, was calculated using the equation percent cytotoxicity = [(cpm of experimental release − cpm of spontaneous release)] / [(cpm of maximum release − cpm of spontaneous release)] × 100. All assays were performed in triplicate.

### Induction of liver metastasis

To induce tumors *in vivo*, Hepa1-6 tumor cells in 0.2 mL of medium 199 (Sigma-Aldrich, St. Louis, MO, USA), at a concentration of 10^7^ cells/mL, were injected into the spleen[19]. C57BL/6J (B6) (H-2b) mice were anesthetized by intraperitoneal injection of xylazine (5 mg/kg body weight) and ketamine (100 mg/kg body weight), and the abdomen was surgically opened. After the spleen was identified, Hepa1-6 tumor cells were slowly injected.

### Isolation of NK cells and adoptive transfer assays

Liver lymphocytes were obtained from wild type B6 mice that had received an intraperitoneal injection of polyinosinic-polycytidylic acid (poly I:C; 150 µg/mouse) (Sigma-Aldrich, St. Louis, MO, USA) 24 h prior to harvesting. Poly I:C activates NK cells primarily by inducing the production of type I (α, β) IFN and IL-12 from a wide variety of cell types [20]. Liver NK cells were then negatively separated using a mouse NK cell isolation kit II (Miltenyi Biotec, Bergisch Gladbach, Germany). The purity of isolated NK cells was assessed by flow cytometry. The purity of the sorted cells was routinely 85%. Three days after injection of the tumor cells, tumor-bearing mice were randomly assigned to either a group treated with 5 × 10^5^ NK cells in 0.2 mL of medium 199 or a control group treated with only medium 199.

### Histological evaluation of metastatic growth in the liver

The mice were sacrificed seven days after tumor cell injection, and the liver was removed and fixed overnight in 10% formalin. For studies using a BZ-8000 microscope (Keyence, Osaka, Japan), 4-µm tissue sections of the liver were stained with HE. The relative areas occupied by the tumors were calculated as the percentage of the total scanned liver area using a BZ-H1M3 image analyzer (Keyence).

### Isolation of NK1.1^-^ CD3^-^ cells and transfer assays

To deplete NK1.1^+^ cells, B6 mice were treated with intraperitoneal injections of anti-NK1.1 antibody (PK136) (200 µg/mouse) 3 d before the isolation of lymphocytes from the liver, spleen, and BM. The anti-NK1.1 antibody (PK136) was prepared in the laboratory using protocols described in a previous study [21]. Next, CD3^-^ cells were purified using a magnetic cell sorting system (Miltenyi Biotec, Bergisch Gladbach, Germany), according to the manufacturer's instructions. For purification of CD3^-^ cells, cell suspensions were incubated with a FITC-labeled mAb specific for CD3e (BD PharMingen, Hamburg, Germany) and then with an anti-FITC mAb coupled to super-paramagnetic microbeads (Miltenyi Biotec, Bergisch Gladbach, Germany). Negative selection columns mounted in a magnetic stand were used to deplete the CD3^+^ cells. The purity of sorted cell populations was confirmed to be >90% using post-sort flow cytometry. RAG-2γ(c) knockout mice, either untreated or irradiated, intravenously received purified NK1.1^−^CD3^−^ liver lymphocytes, NK1.1^−^CD3^−^ splenic lymphocytes, or NK1.1^−^CD3^−^ BM lymphocytes.

### Statistical analysis

Unpaired Student’s *t*-tests, the nonparametric Mann-Whitney U test, and ANOVA were performed to compare differences between the two independent groups. p < 0.05 was considered statistically significant. Values are expressed as the mean ± standard deviation (SD). JMP 11 software (SAS Institute Inc., Cary, NC, USA) was used for all calculations.

## Results

### Hepatic irradiation did not affect intra-hepatic lymphocytes

**Fig 1:**
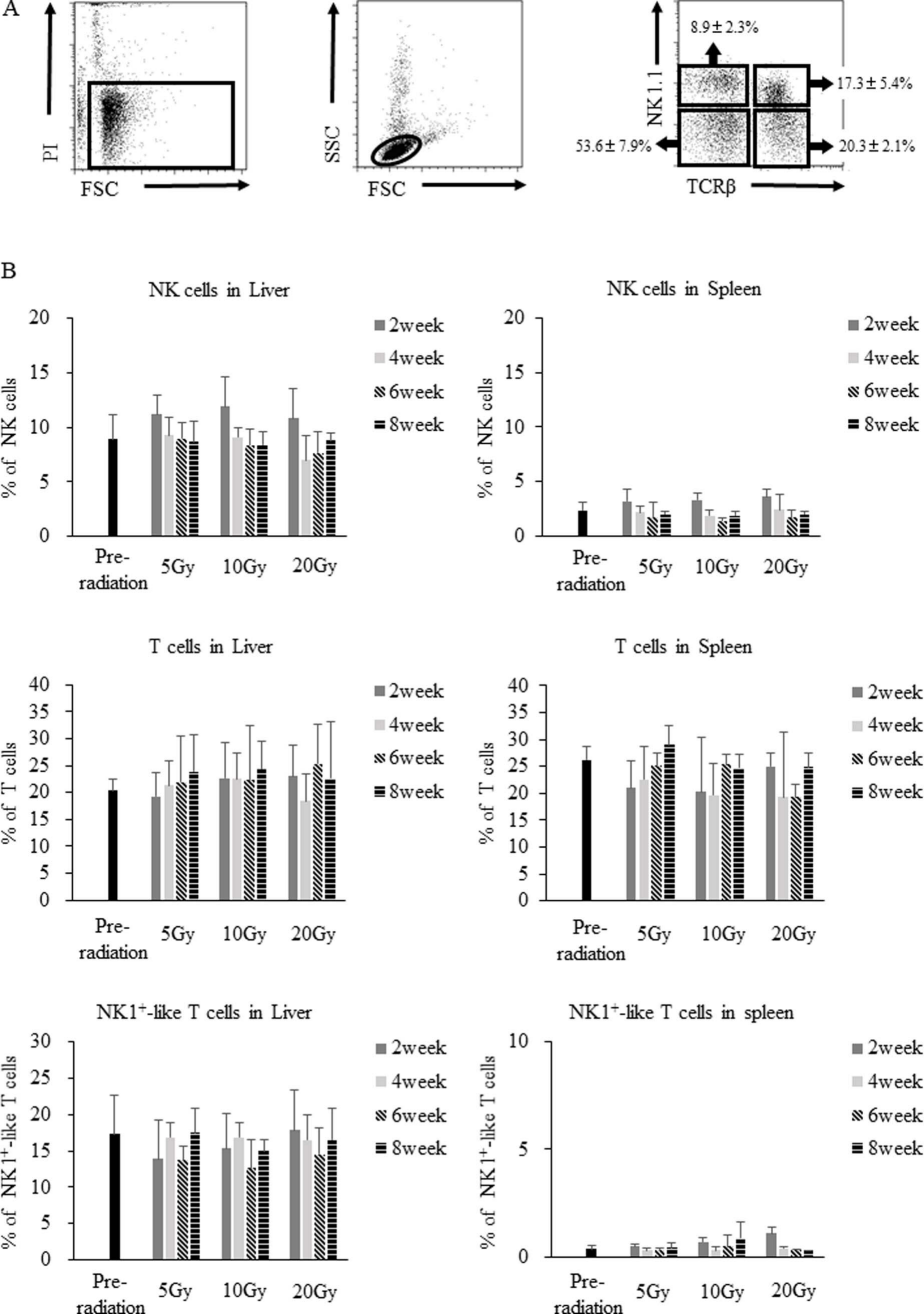
Time course analysis of liver and splenic lymphocytes. Liver and splenic lymphocytes were isolated from control or hepatic irradiated B6 mice. (A) Liver and splenic lymphocytes were stained with anti-NK1.1, anti-TCRβ, and PI. Representative flow panels show the percentages of NK1.1^+^TCRβ^−^ NK cells, NK1.1^+^TCRβ^+^ NK1^+^-like T cells, and NK1.1^−^TCRβ^+^ T cells among the live lymphocyte population in the liver. (B) The overall proportions of NK cells, NK1^+^-like T cells, and T cells among the live lymphocyte population in the liver and spleen did not change in the 2 months after hepatic irradiation (n = 4). Data are expressed as the mean ± SD. Statistical differences were assessed using the nonparametric Mann-Whitney U test.

We established a murine hepatic irradiation model based on previously published reports that assessed the effects of whole liver irradiation in rats [17, 22]. S1A Fig summarizes the AST and ALT plasma levels in each group of mice. After hepatic irradiation, AST and ALT plasma levels tended to increase. However, microscopy and HE staining showed no significant changes for 8 weeks after hepatic irradiation (S1B Fig). The NK cell population was examined in mice with hepatic irradiation, using single-fraction doses of 5 Gy, 10 Gy, or 20 Gy. We observed that the overall numbers and proportions of total NK, NK1^+^-like T cells [23], and T cells in the livers and spleens of the mice did not change in the two months after hepatic irradiation when compared to the baseline before hepatic irradiation (Fig 1A and 1B). To analyze the influence of contamination of γδT cells contained in the TCRβ^-^NK1.1^+^ population, we checked the presence of γδT cells in liver NK cells. We could detect only 1.8 ± 0.3% γδT cells among whole liver NK cells and 1.7 ± 0.2% γδT cells among lrNK cells (S2 Fig).

### The reduction in DX5^–^TRAIL^+^ lrNK cells after irradiation persisted for two months after irradiation

**Fig 2:**
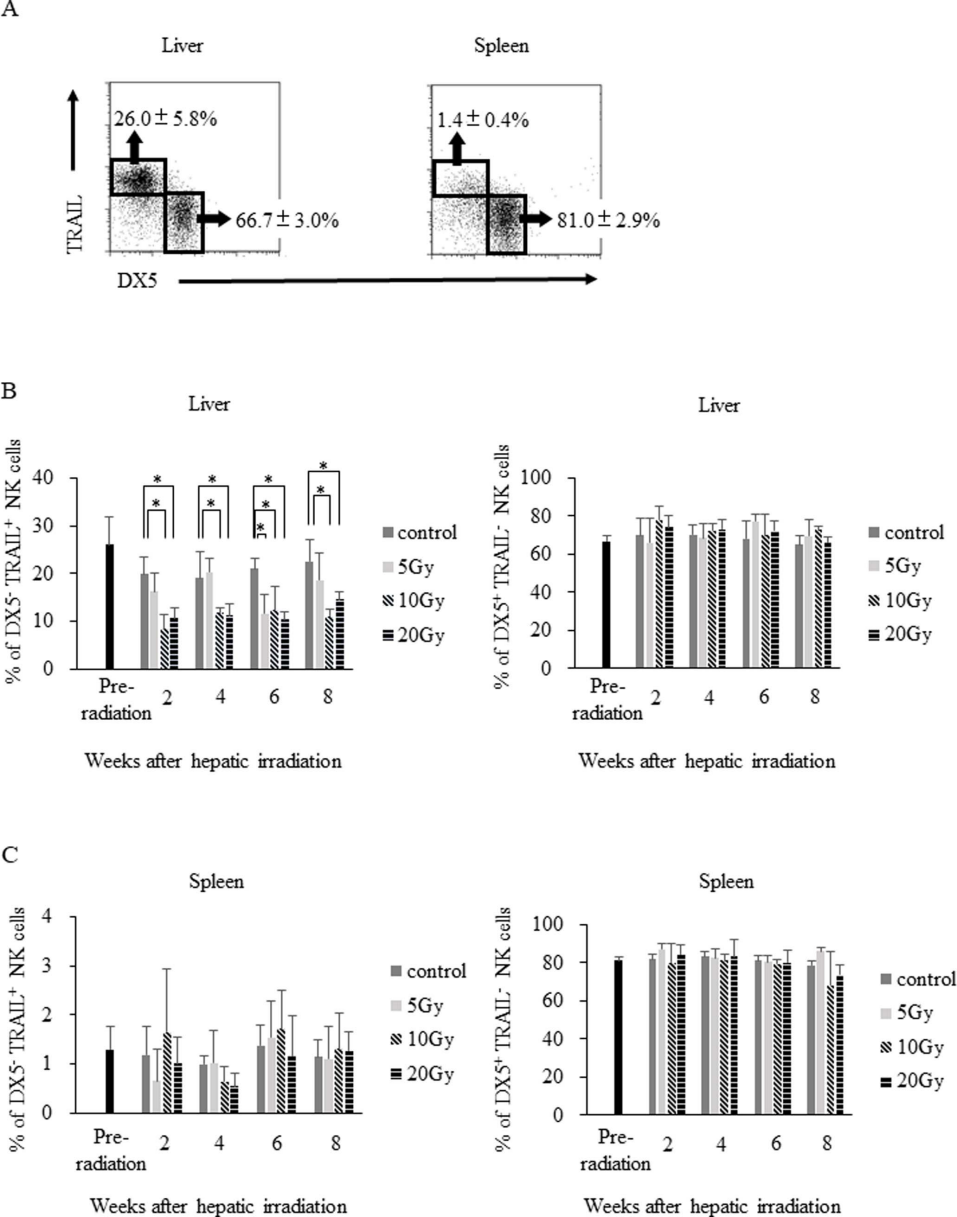
Hepatic irradiation decreases the proportion of DX5^–^TRAIL^+^ lrNK cells for up to two months. (A) The liver and splenic lymphocytes were stained with anti-NK1.1, anti-TCRβ, and PI. NK1.1^+^TCRβ^−^ NK cells were then gated for the analysis of other markers. NK cells were divided into DX5^–^TRAIL^+^ lrNK cells and DX5^+^TRAIL^−^ cNK cells. (B) After hepatic irradiation, the lrNK cell population was significantly decreased, whereas the cNK cell population did not change, in livers irradiated with 10 Gy or 20 Gy when compared to sham-operation mice (n = 4). (C) The populations of DX5^−^TRAIL^+^ lrNK cells and DX5^+^ TRAIL^−^ cNK cells in the spleen did not change after hepatic irradiation in the 5 Gy, 10 Gy, and 20 Gy groups (n = 4). Data are expressed as the mean ± SD. Statistical differences were assessed using the nonparametric Mann-Whitney U test (*p < 0.05).

We next compared the expression of various surface markers on hepatic NK cells, showing that the proportion of lrNK cells (defined as DX5^–^TRAIL^+^) significantly decreased in the mice exposed to 10 Gy or 20 Gy when compared to the sham-operation group. LrNK cells were maintained in mice irradiated with 5 Gy. This showed that hepatic irradiation specifically decreased the amount of DX5^–^TRAIL^+^ lrNK cells in a dose-dependent manner (Fig 2A and 2B). In contrast, the frequency of DX5^-^TRAIL^-^NK cells significantly increased in mice exposed to 10 Gy or 20 Gy when compared to the sham-operated group (S3 Fig). Further, the phenotype of splenic NK cells did not change in either group (Fig 2C). Taken together, these data suggest that hepatic irradiation strongly affected the maintenance of DX5^–^TRAIL^+^ lrNK cells.

### Hepatic irradiation dampened the cytotoxic activity of hepatic NK cells against hepatoma cells

**Fig 3:**
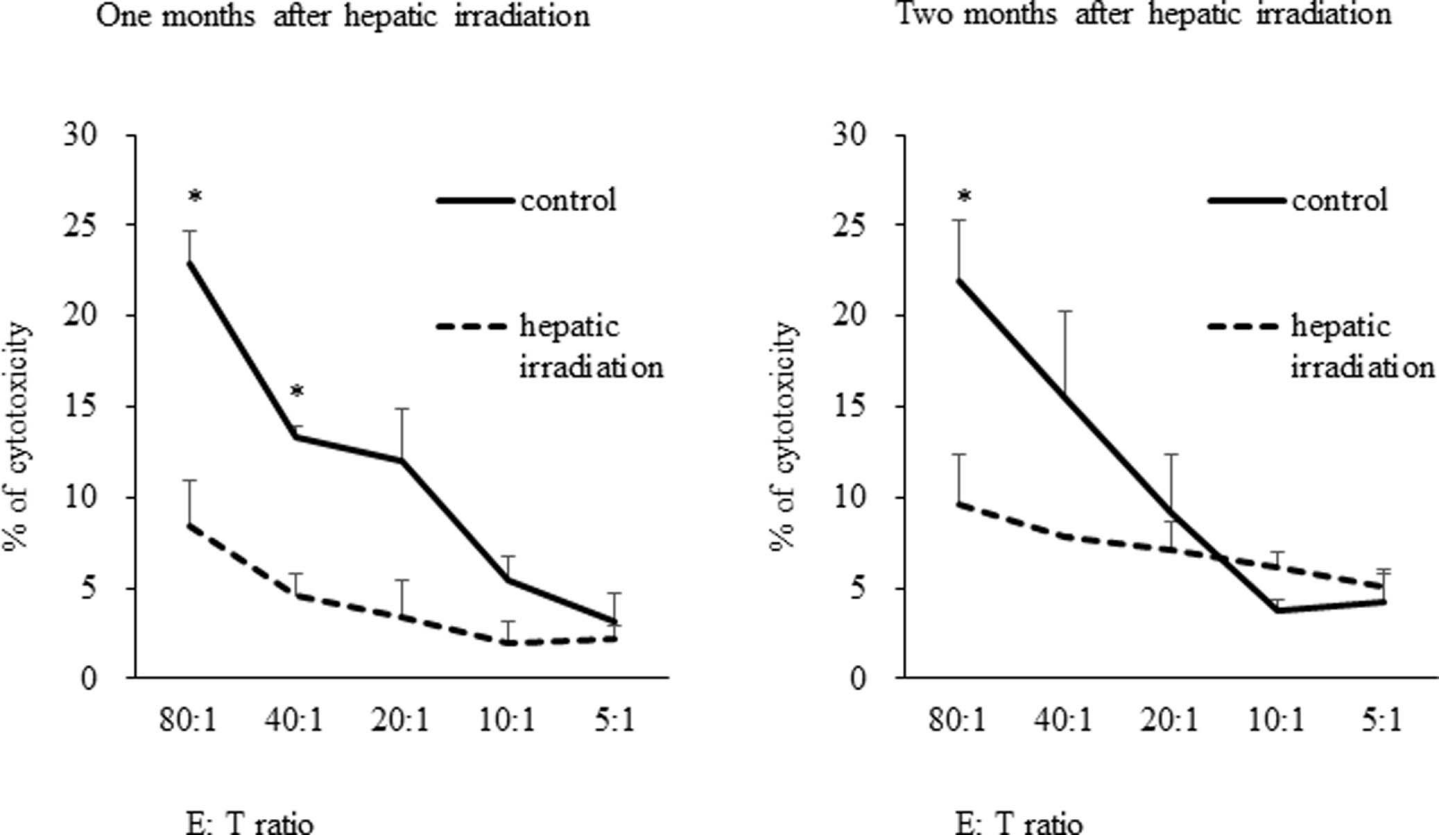
Hepatic irradiation decreases the cytotoxic activities of liver lymphocytes. The cytotoxicity of liver lymphocytes after hepatic irradiation using single-fraction doses of 10 Gy was decreased at both one (left) and two (right) months after irradiation. Freshly isolated liver lymphocytes after sham operation were used as the control. Data are expressed as the mean ± SD. (n = 4 mice per group). Statistical differences were assessed using ANOVA (*p < 0.05).

We previously reported that partial hepatectomy decreased the expression of TRAIL in lrNK cells and decreased their cytotoxic potential [13]. We therefore examined the cytotoxic activity of liver-derived lymphocytes in mice after hepatic irradiation. As shown in Fig 3, freshly isolated liver lymphocytes possessed potent cytotoxicity against TRAIL-sensitive hepatoma Hepa1-6 cells. However, liver lymphocytes isolated from mice that had received hepatic irradiation one or two months prior were unable to mediate cytotoxicity (Fig 3). Next, we examined the cytotoxic activity of isolated NK cells among liver lymphocytes after hepatic irradiation. Freshly isolated liver NK cells possessed potent cytotoxicity against Hepa1-6 cells. However, isolated liver NK cells that had received hepatic irradiation one month prior were unable to mediate cytotoxicity (S4 Fig).

### Adoptive transfer of lrNK cells inhibited liver metastasis in hepatic-irradiated mice

**Fig 4:**
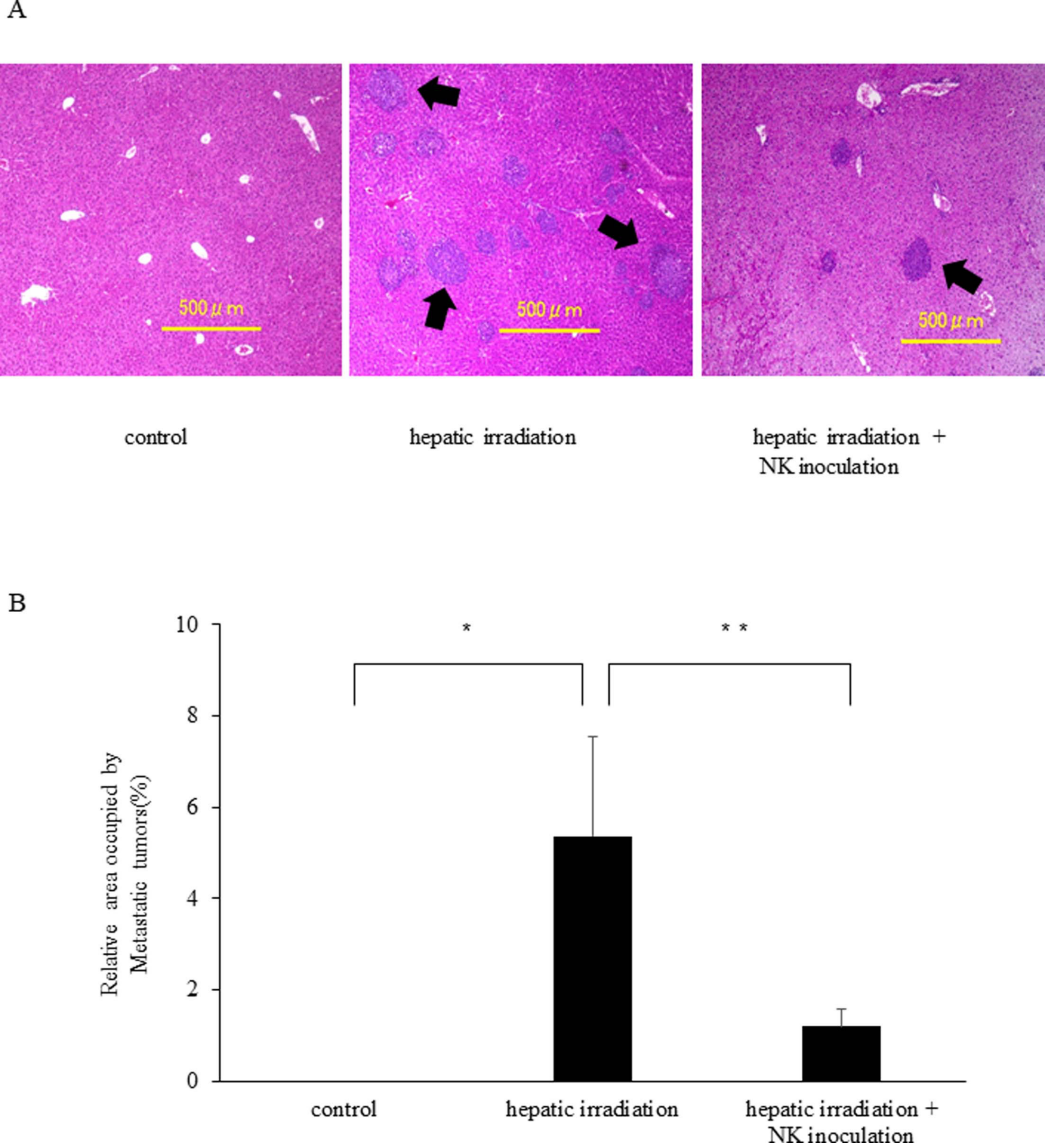
Hepatic irradiation increases hepatic metastasis and adoptive transfer of activated NK cells inhibits this effect. (A) Representative histopathological findings of liver specimens (stained with H&E). Specimens are shown from the control group (left), hepatic irradiated group (middle), and the group that received irradiation followed by NK cell transplantation (right). Arrows indicate metastatic tumors. (B) Seven days before splenic injection of Hepa1-6, mice underwent hepatic irradiation using single-fraction doses of 10 Gy or did not undergo hepatic irradiation as the control group and were injected with tumor cells on Day 0. On Day 3, the hepatic irradiation + NK inoculation group (n = 5) received an intravenous injection of 5 × 10^5^ liver NK cells, whereas control group (n = 5) and the hepatic irradiation group (n = 7) received medium alone. The proliferation of hepatoma cells was promoted but the adoptive transfer of activated liver NK cells inhibited liver metastasis. Data are represented as the mean ± SD. Statistical differences were assessed using unpaired Student’s *t*-test (* p < 0.05 for control vs. hepatic irradiation; ** p < 0.05 for hepatic irradiation vs. hepatic irradiation + NK inoculation mice).

We next analyzed whether the reduced activity of liver NK cells after hepatic irradiation also promoted the proliferation of hepatoma cells *in vivo*. As shown in Fig 4A, additional liver tumors were detected after hepatic irradiation in mice. Conversely, fewer liver tumors were detected in control mice that were not irradiated. To further analyze the anti-tumor effects of lrNK cells, we investigated whether adoptive transfer of lrNK cells could reconstitute these defensive activities after hepatic irradiation in mice. Three days after administering Hepa1-6 cells to hepatic-irradiated mice, we intravenously administered NK cells extracted from the livers of syngeneic mice. Mice receiving liver NK cells showed significantly less metastasis one week after inoculation (Fig 4A and 4B).

### Hepatic irradiation inhibited the development of precursors to lrNK cells in the liver

**Fig 5:**
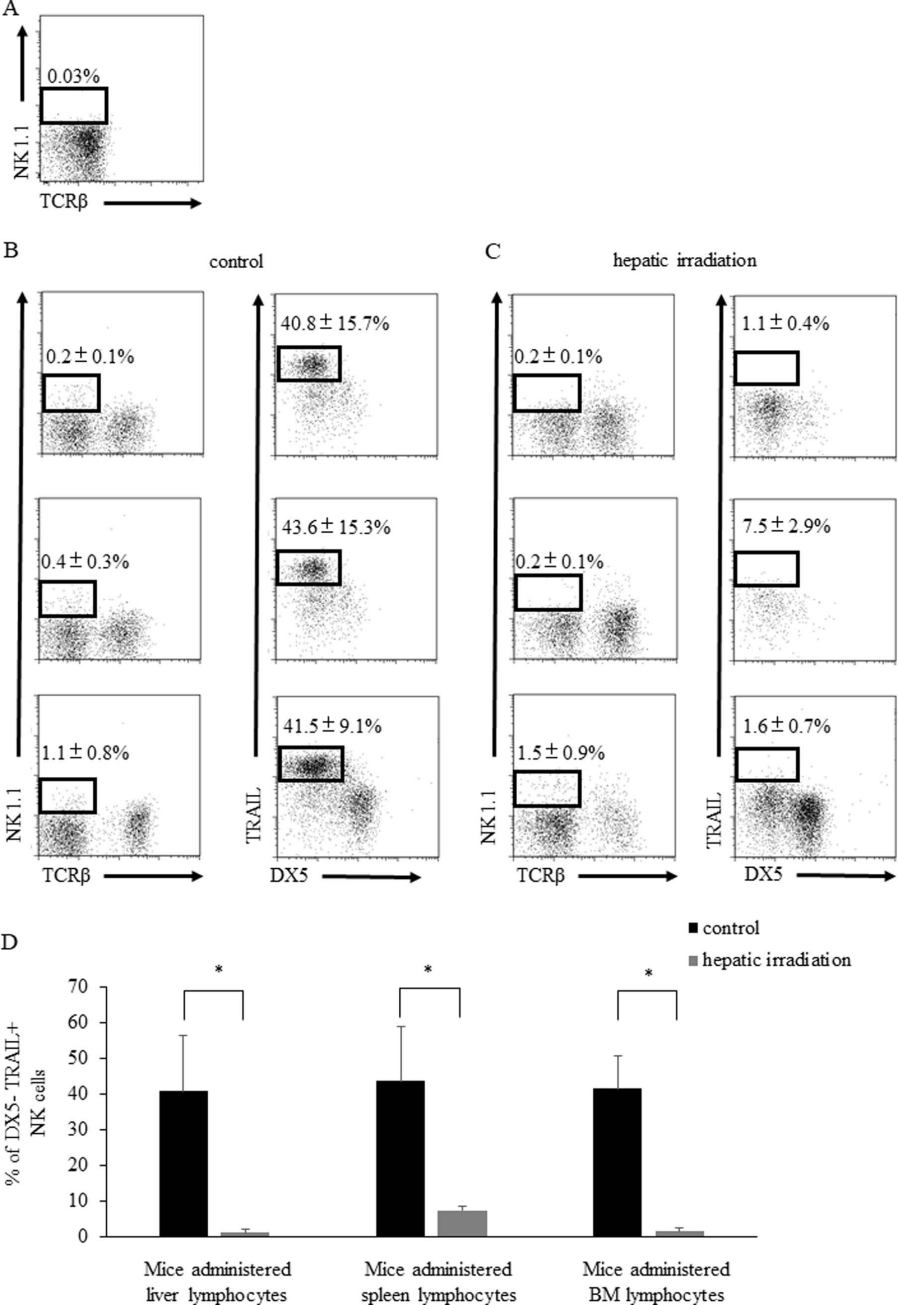
DX5^–^TRAIL^+^ NK cells were generated from non-T, non-NK precursors (CD3^−^NK1.1^−^ cells) isolated from liver lymphocytes, splenic lymphocytes, and BM cells in control mice, but not from mice that received liver irradiation. (A) Liver lymphocytes of RAG-2γ(c) knockout mice were stained with anti-NK1.1, anti-TCRβ, and PI. Representative flow panels show the percentages of NK1.1^+^TCRβ− fractionation among the live lymphocyte population in the liver. (B) Representative flow cytometry plots of DX5^–^TRAIL^+^ lrNK cells isolated from the liver in CD3^–^NK1.1^–^ liver lymphocyte-administered (upper), CD3^–^NK1.1^–^ splenic lymphocyte-administered (middle), and CD3^–^NK1.1^–^ BM lymphocyte-administered (lower) mice, gated on the total NK1.1^+^ TCRβ^−^ cell population (n = 5). (C) Representative flow cytometry plots of DX5^–^TRAIL^+^ lrNK cells isolated from the liver in CD3^–^NK1.1^–^ liver lymphocyte-administered (upper), CD3^–^NK1.1^–^ splenic lymphocyte-administered (middle), and CD3^–^NK1.1^–^ BM lymphocyte-administered (lower) mice that received hepatic irradiation, gated on the total NK1.1^+^ TCRβ^−^ cell population (n = 5). (D) The proportion of DX5^-^ TRAIL^+^ NK cells in control mice was significantly higher than that in mice with hepatic irradiation in the CD3^–^NK1.1^–^ liver lymphocyte-administered group (left), CD3^–^NK1.1^–^ splenic lymphocyte-administered group (middle), and CD3^–^NK1.1^–^ BM lymphocyte-administered group (right). Data are presented as the mean ± SD. Statistical differences were assessed using unpaired Student’s *t*-test (* p < 0.05 for control vs. hepatic irradiation mice).

As our data suggest that hepatic irradiation plays a negative role in the maintenance of DX5^−^TRAIL^+^ lrNK cells in the liver, we next assessed if there was an effect on their development. However, the identities of the precursors of lrNK cells are controversial, with both the liver [15] and BM [24] previously reported to be the source of these cells. To investigate the precursors of lrNK cells, we transferred purified non-T, non-NK, and non- NK1^+^-like T cell precursors (CD3^−^NK1.1^−^ cells) from liver lymphocytes, splenic lymphocytes, and bone marrow cells to RAG-2γ(c) knockout mice (S5 and 5A Figs). Surprisingly, DX5^−^TRAIL^+^ NK cells were generated in the recipient liver one month after transfer into each of the groups (Fig 5B). This demonstrated that the precursors of the hepatic DX5^–^TRAIL^+^ lrNK cell subset exist in the liver, spleen, and BM. The discovery that the precursors of hepatic NK cells were present in the spleen and BM, in addition to the liver, and that hepatic irradiation abolished the DX5^-^TRAIL^+^ NK cell population associated with the liver environment, suggested that hepatic irradiation may alter NK cell development. To investigate this possibility, we transferred purified non-T, non-NK, and non- NK1^+^-like T cells precursors (CD3^–^NK1.1^–^ cells) from liver lymphocytes, splenic lymphocytes, or BM cells to RAG-2γ(c) knockout mice that had received hepatic irradiation one week prior to the experiment. Although the precursor cells from liver, spleen, and BM were adoptively transferred to hepatic irradiated mice, DX5^–^TRAIL^+^ lrNK cells were still not generated in the recipient liver up to one month after transfer (Fig 5C and 5D). These results suggest that hepatic irradiation abolished the precursor cells in the liver and altered the environment in which lrNK cells differentiated.

## Discussion

In this study, we show that hepatic irradiation specifically eliminated the DX5^–^TRAIL^+^ lrNK cell population. This situation was maintained for at least two months after the hepatic irradiation procedure. Using a hepatoma cell line, we found that hepatic irradiation also reduced the cytotoxic activity of NK cells in the liver, promoting tumor growth. Furthermore, adoptive transfer of precursor cells could not restore the DX5^–^TRAIL^+^ lrNK cell population in the liver lost after hepatic irradiation. Our data suggest that hepatic irradiation changes the developmental environment of the liver, preventing precursor cell differentiation into lrNK cells.

The effects of irradiation on the immune system have been demonstrated in several tissues. For example, partial irradiation of rat lungs increased the expression of TNF, IL-6, and TGF-β [25]. Local radiotherapy also promotes the generation of tumor-specific effector T cells via dendritic cell activation [26]. In a hepatic irradiation rat model, the expression of alpha smooth muscle actin (α-SMA) and transforming growth factor-beta (TGF-β) was also shown to increase as a result of liver fibrosis [17]. In our current study, hepatic irradiation was found to decrease only the amount of DX5^–^TRAIL^+^ lrNK cells, and the numbers of conventional NK cells, NK1^+^-like T cells, and T cells in the liver remained unchanged. Furthermore, the frequency of DX5^-^TRAIL^-^NK cells significantly increased after hepatic irradiation. This DX5^-^TRAIL^-^ population belongs to a more immature population named NK cell-committed precursors [27]. To summarize these data, hepatic irradiation decreased the proportion of lrNK cells, and as a result, the amount of immature NK precursor cells increased in the liver. In addition, previous reports have shown that NK cell accumulation is dependent on certain cytokines and chemokines, such as IFN-γ and CXCR3 ligands [28], and our own recent findings suggest that CXCL9 mRNA levels are significantly decreased in the liver after hepatectomy [29]. Together with our present data, these studies suggest that the microenvironment change elicited by irradiation affects only lrNK cells.

LrNK cells are defined as being DX5^–^, with elevated levels of TRAIL, and are capable of inducing cell death in TRAIL-sensitive target cells [15, 30, 31]. Recently, different transcriptional networks have been shown to control the development of lrNK cells and conventional NK cells, suggesting that lrNK cells are distinct from conventional NK cells that develop from BM progenitors [32–34]. Indeed, it has been shown that the transcription factor T-bet is required for early development of lrNK cells but has only a moderate effect on conventional NK cell development. In contrast, lrNK cells express eomesodermin (Eomes) at low levels compared to conventional NK cells. Eomes is important for conventional NK cell development but does not impact lrNK cells [33, 35]. We have previously shown that lrNK cells play important roles in anti-tumor immunity in the liver, especially after a hepatectomy [8, 13]. It has been reported that after adoptive transfer, lrNK cells migrate only to the liver [15].We have already reported that injected hepatic NK cells could migrate into the liver via the CXCL9-CXCR3 pathway and could attack tumor cells in the liver [13, 29]. Our results show that hepatic irradiation promoted tumor growth, and adoptive transfer of liver NK cells inhibited tumor growth in mice that underwent hepatic irradiation. Our observation that hepatic irradiation completely removed the lrNK cell population implies that such irradiation weakens anti-tumor immunity in the liver.

There are, however, some limitations to the present study. First, the observation period was short and limited to two months after irradiation. Therefore, the long-term effect of hepatic irradiation on lrNK cells remains unknown. However, the dampening of NK cell-mediated immunity in the liver likely affects tumor growth shortly after hepatic irradiation, limiting the impact of using a short timescale. We therefore believe that the phenomenon we have observed is important in clinical situations. Second, the underlying mechanisms of hepatic irradiation on lrNK cells remain unclear and we could not identify any specific mechanism in the study. This was because many cytokines and chemokines change in the liver following hepatic irradiation, making relationships difficult to ascertain. Third, whole liver irradiation is not a general treatment. Local hepatic irradiation is focused on where the tumor masses are. Such local irradiation might not be associated with persistent elimination of lrNK cells. Rather, local irradiation could reduce the size of tumors, thereby enhancing the killing of tumor cells by NK cells surrounding the irradiated area. Fourth, the frequency of NK cells that migrated into the liver was very small. The transferred fraction did not contain NK1.1^+^ cells because this population was depleted using monoclonal antibodies. Hepatic irradiation significantly decreased the number of lrNK cells and increased the frequency of immature NK precursors. These results indicate that hepatic irradiation could suppress differentiation from immature NK precursors to lrNK cells in the liver. Additionally, there has been no evidence indicating that lrNK cells are particularly sensitive to radiation. Furthermore, the sensitivity and proportion of the liver lymphocyte fraction in the liver is likely different between mice and humans. These issues should be assessed in future studies.

In conclusion, hepatic irradiation eliminated the DX5^–^TRAIL^+^ lrNK cell population, and this situation was maintained for at least two months after irradiation. Furthermore, precursor cells failed to differentiate into lrNK cells after hepatic irradiation. These data highlight the effects of such treatment on immune cells in the liver and suggest possible new avenues of research that may contribute to improving the outcome for patients with hepatobiliary malignancies.

## Supporting information

S1 Fig Time course analysis of blood test for liver function and pathological changes in liver. (A) After hepatic irradiation, AST and ALT plasma levels in mice that received hepatic irradiation tended to be higher compared to those of sham-operated mice for up to two months. (n = 4). Data are expressed as the mean ± SD. Statistical differences were assessed using the nonparametric Mann-Whitney U test. (*p < 0.05). (B) Representative histopathological findings of liver specimens (stained with H&E). Specimens are shown from pre-irradiation mice (left), 4 weeks after hepatic irradiation (middle), and 8 weeks after hepatic irradiation (right).
(TIF)

S2 Fig γδT cells in liver NK cell analysis. Liver lymphocytes were isolated from B6 mice. (A) Liver lymphocytes were stained with anti-NK1.1, anti- TCRβ and 7-AAD. NK1.1^+^ TCRβ^-^NK cells were then gated for the analysis of other markers. We defined γδT cells as CD3^–^TCRγδ^+^ cells. Representative flow panels show the percentages of γδT cells among liver NK cells. (B)DX5^–^TRAIL^+^ lrNK were then gated for the analysis of γδT cells. Representative flow panels show the percentages of γδT cells among lrNK cells (n = 3). Data are expressed as the mean ± SD.
(TIF)

S3 Fig Hepatic irradiation increases the proportion of DX5^–^TRAIL^-^ NK cells for up to two months. After hepatic irradiation, DX5^–^TRAIL^-^ NK cell population was significantly increased in livers irradiated with 10 Gy or 20 Gy when compared to those of sham-operated mice (n = 4). Data are expressed as the mean ± SD. Statistical differences were assessed using the nonparametric Mann-Whitney U test (*p < 0.05).
(TIF)

S4 Fig Hepatic irradiation decreases the cytotoxic activities of liver NK cells. The cytotoxicity of isolated NK cells in liver lymphocytes after hepatic irradiation using single-fraction doses of 10 Gy was decreased at one month after irradiation. Freshly isolated liver NK cells after sham operation were used as the control. Data are expressed as the mean ± SD. (n = 15 mice per group). Statistical differences were assessed using ANOVA (*p < 0.05).
(TIF)

S5 Fig Phenotype of transferred cells. Representative flow cytometry plots of CD3 and NK1.1 depleted liver lymphocytes extracted from wild-type B6 mice (left), CD3 and NK1.1 depleted splenic lymphocytes extracted from wild-type B6 mice (middle), and CD3 and NK1.1 depleted BM lymphocytes extracted from wild-type B6 mice (right). Representative flow panels show the percentages of NK1.1^+^TCRβ^−^ NK cells.
(TIF)
