# Supplementary figures and images for: Hepatic irradiation persistently eliminates liver resident NK cells
Source: PLoS One. 2018 Jun 13;13(6):e0198904. doi: 10.1371/journal.pone.0198904 (PMC5999234; doi:10.1371/journal.pone.0198904)

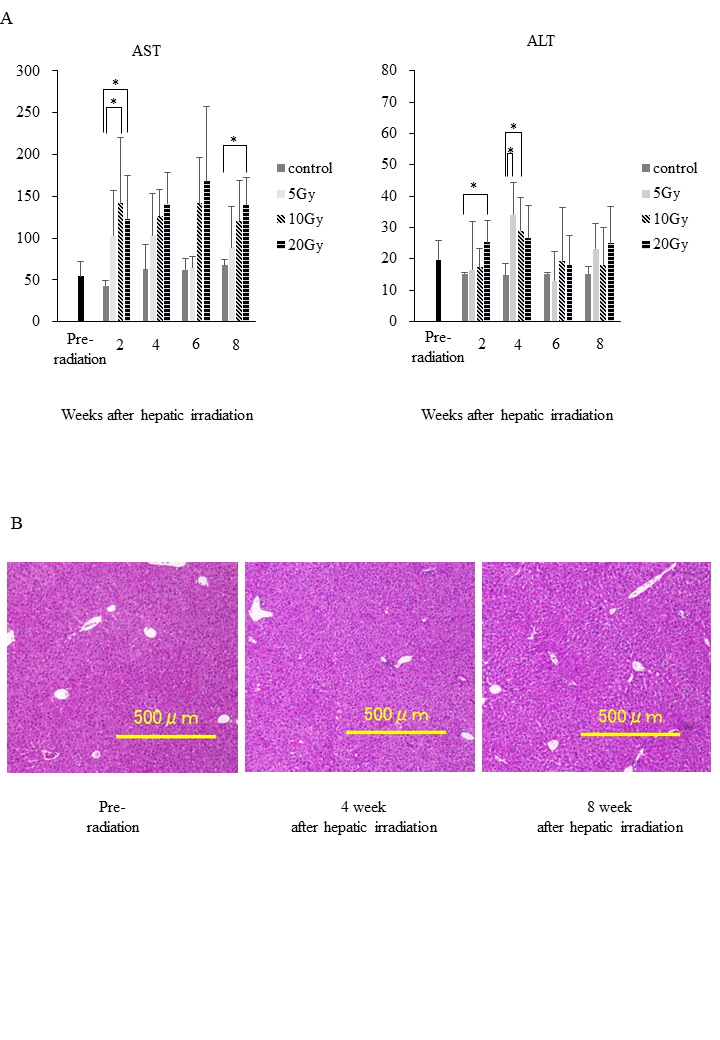

Supplement: S1 Fig — (A) After hepatic irradiation, AST and ALT plasma levels in mice that received hepatic irradiation tended to be higher compared to those of sham-operated mice for up to two months. (n = 4). Data are expressed as the mean ± SD. Statistical differences were assessed using the nonparametric Mann-Whitney U test. (*p < 0.05). (B) Representative histopathological findings of liver specimens (stained with H&E). Specimens are shown from pre-irradiation mice (left), 4 weeks after hepatic irradiation (middle), and 8 weeks after hepatic irradiation (right). (TIF) [file pone.0198904.s001.tif]

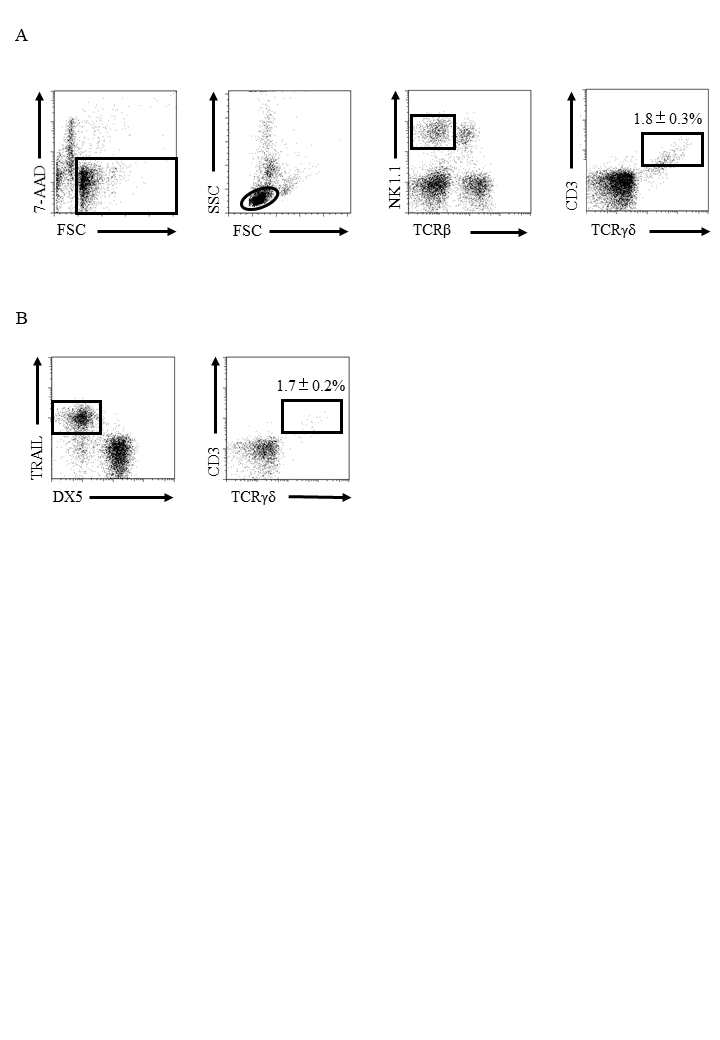

Supplement: S2 Fig — Liver lymphocytes were isolated from B6 mice. (A) Liver lymphocytes were stained with anti-NK1.1, anti- TCRβ and 7-AAD. NK1.1+ TCRβ-NK cells were then gated for the analysis of other markers. We defined γδT cells as CD3–TCRγδ+ cells. Representative flow panels show the percentages of γδT cells among liver NK cells. (B)DX5–TRAIL+ lrNK were then gated for the analysis of γδT cells. Representative flow panels show the percentages of γδT cells among lrNK cells (n = 3). Data are expressed as the mean ± SD. (TIF) [file pone.0198904.s002.tif]

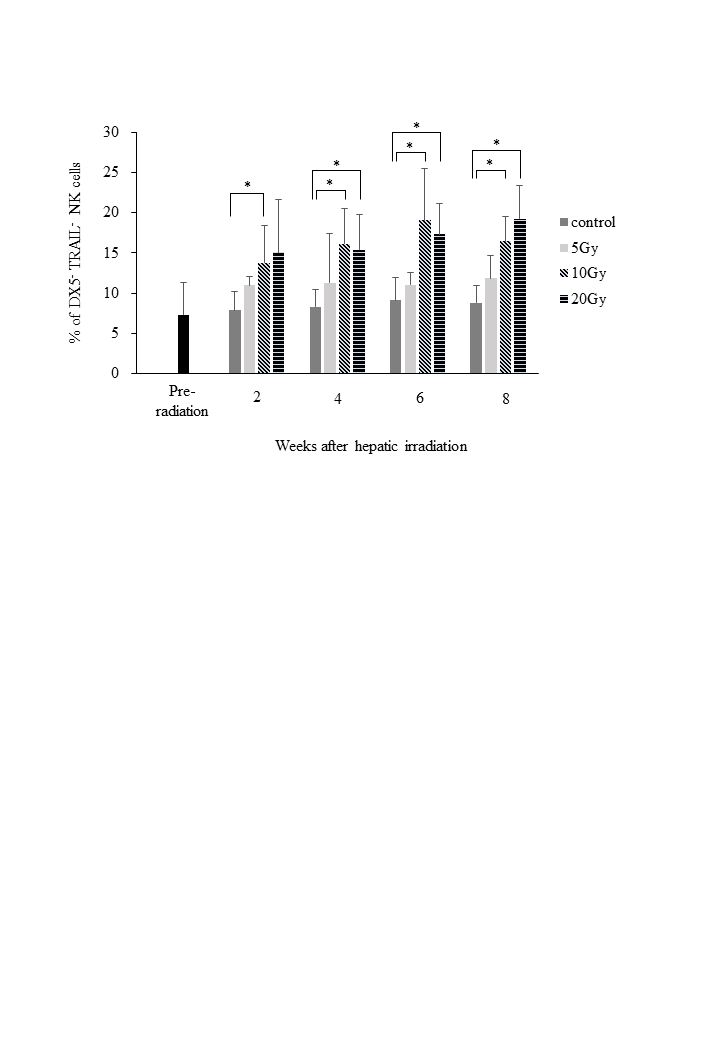

Supplement: S3 Fig — After hepatic irradiation, DX5–TRAIL- NK cell population was significantly increased in livers irradiated with 10 Gy or 20 Gy when compared to those of sham-operated mice (n = 4). Data are expressed as the mean ± SD. Statistical differences were assessed using the nonparametric Mann-Whitney U test (*p < 0.05). (TIF) [file pone.0198904.s003.tif]

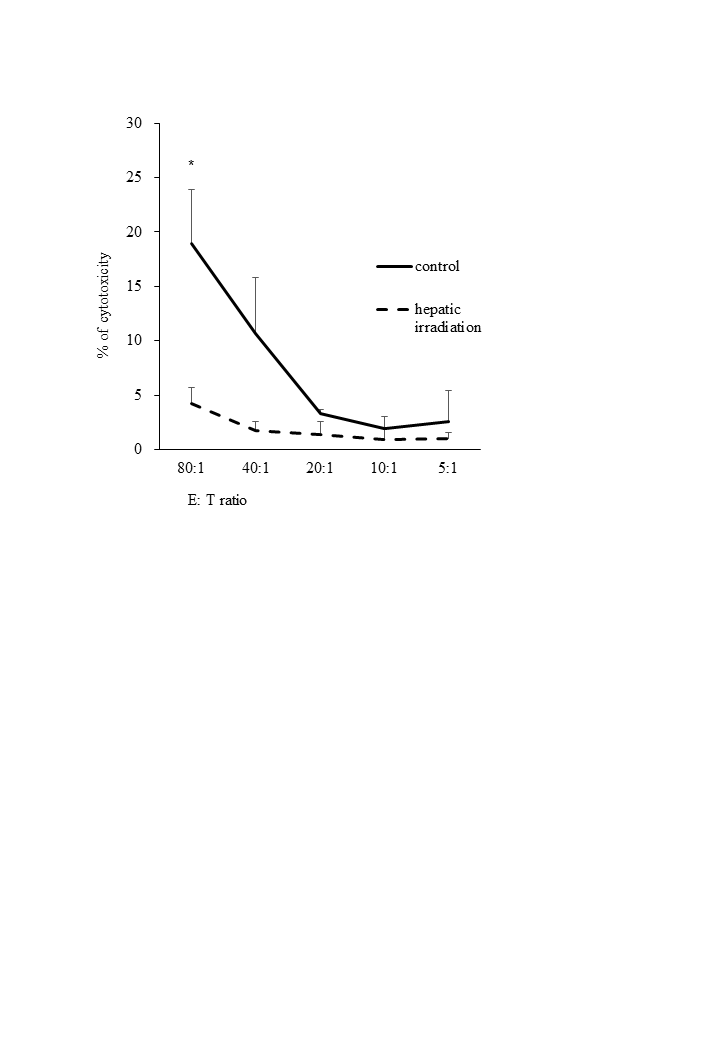

Supplement: S4 Fig — The cytotoxicity of isolated NK cells in liver lymphocytes after hepatic irradiation using single-fraction doses of 10 Gy was decreased at one month after irradiation. Freshly isolated liver NK cells after sham operation were used as the control. Data are expressed as the mean ± SD. (n = 15 mice per group). Statistical differences were assessed using ANOVA (*p < 0.05). (TIF) [file pone.0198904.s004.tif]

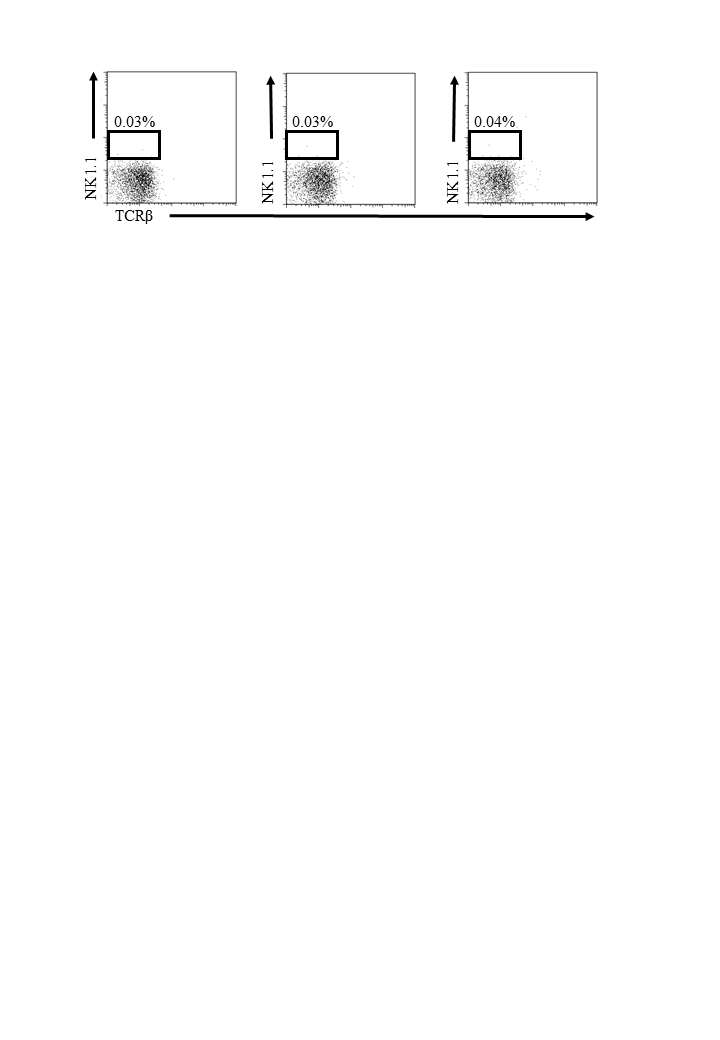

Supplement: S5 Fig — Representative flow cytometry plots of CD3 and NK1.1 depleted liver lymphocytes extracted from wild-type B6 mice (left), CD3 and NK1.1 depleted splenic lymphocytes extracted from wild-type B6 mice (middle), and CD3 and NK1.1 depleted BM lymphocytes extracted from wild-type B6 mice (right). Representative flow panels show the percentages of NK1.1+TCRβ− NK cells. (TIF) [file pone.0198904.s005.tif]
